# Bacterial Longevity Requires Protein Synthesis and a Stringent Response

**DOI:** 10.1128/mBio.02189-19

**Published:** 2019-10-15

**Authors:** Liang Yin, Hongyu Ma, Ernesto S. Nakayasu, Samuel H. Payne, David R. Morris, Caroline S. Harwood

**Affiliations:** a Department of Microbiology, University of Washington, Seattle, Washington, USA; b Department of Environmental Science and Engineering, Xi’an Jiaotong University, Xi’an, People’s Republic of China; c Biological Sciences Division, Pacific Northwest National Laboratory, Richland, Washington, USA; d Department of Biology, Brigham Young University, Provo, Utah, USA; e Department of Biochemistry, University of Washington, Seattle, Washington, USA; California Institute of Technology

**Keywords:** *Rhodopseudomonas palustris*, growth arrest, translation, longevity, stringent response, ribosomes

## Abstract

Gram-negative bacteria in infections, biofilms, and industrial settings often stop growing due to nutrient depletion, immune responses, or environmental stresses. Bacteria in this state tend to be tolerant to antibiotics and are often referred to as dormant. Rhodopseudomonas palustris, a phototrophic alphaproteobacterium, can remain fully viable for more than 4 months when its growth is arrested. Here, we show that protein synthesis, specific proteins involved in translation, and a stringent response are required for this remarkable longevity. Because it can generate ATP from light during growth arrest, R. palustris is an extreme example of a bacterial species that will stay alive for long periods of time as a relatively homogeneous population of cells and it is thus an excellent model organism for studies of bacterial longevity. There is evidence that other Gram-negative species also continue to synthesize proteins during growth arrest and that a stringent response is required for their longevity as well. Our observations challenge the notion that growth-arrested cells are necessarily dormant and metabolically inactive and suggest that such bacteria may have a level of metabolic activity that is higher than many would have assumed. Our results also expand our mechanistic understanding of a crucial but understudied phase of the bacterial life cycle.

## Abstract (precis)

We are surrounded by bacteria, but they do not completely dominate our planet despite the ability of many to grow extremely rapidly in the laboratory. This has been interpreted to mean that bacteria in nature are often in a dormant state. We investigated life in growth arrest of Rhodopseudomonas palustris, a proteobacterium that stays alive for months when it is not growing. We found that cells were metabolically active, and they continued to synthesize proteins and mounted a stringent response, both of which were required for their longevity. Our results suggest that long-lived bacteria are not necessarily inactive but have an active metabolism that is well adjusted to life without growth.

## INTRODUCTION

Bacteria in nature often exist in viable but nongrowing states without forming differentiated structures like spores (1, 2), but this crucial phase of the bacterial life cycle is underexplored. Factors that limit cell replication include nutrient limitation, competition within microbial communities, and stresses like oxygen depletion and extreme temperatures (3–5). Several bacterial pathogens, including Vibrio cholerae, Pseudomonas aeruginosa, and Burkholderia pseudomallei, can survive for months and even years in a growth-arrested state in distilled water, sterilized seawater, or basal salts medium in a laboratory setting (6–9). In keeping with this, drinking water is a known reservoir for bacterial pathogens (10). Nongrowing pathogenic bacteria also occur in human infections and in this form present challenges for treatment because antibiotics tend to target processes such as cell wall biosynthesis or DNA replication that are active in growing, but not nongrowing, cells. One example of a bacterium that is recalcitrant to antibiotic treatment *in vivo* is Mycobacterium tuberculosis, which forms latent infections that persist over many years in the absence of overt signs of growth (11). Then there are bacterial antibiotic persisters, a small subpopulation of nongrowing or slowly growing cells that develop in some bacterial infections and are tolerant to antibiotic treatment (12–14).

Studying nongrowing bacteria is also important in environmental, industrial, and other nonmedical contexts. For example, nongrowing bacteria are excellent biocatalysts because they can convert substrates that might be used for growth to value-added products. In past work, we have studied the production of hydrogen gas, a biofuel, by the phototrophic alphaproteobacterium Rhodopseudomonas palustris. When illuminated to allow ATP synthesis by photophosphorylation, nongrowing cells diverted cell resources to hydrogen gas production and generated 300% more hydrogen gas than did growing cells (15). We subsequently determined changes in metabolic fluxes and biomass composition that accounted for the diversion of electrons to hydrogen gas in growth-arrested cells (15). We also found that nongrowing R. palustris cells embedded in thin strips of latex film remained viable and produced hydrogen gas for over 4 months (16).

**FIG 1:**
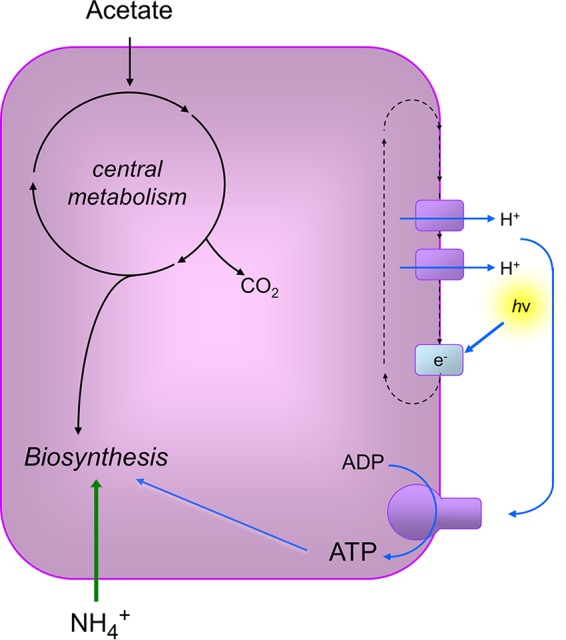
Metabolism of R. palustris during anaerobic growth in light. Acetate was used as the carbon source and ammonium as the nitrogen source for growth in this study. R. palustris generates ATP and proton motive force from light energy by cyclic photophosphorylation. Energy generation and carbon use are independent from each other. In this study, growth arrest occurred when R. palustris cells had used all available acetate. In this circumstance, cells can continue to generate ATP and proton motive force from light.

These results prompted us to develop R. palustris as a model for studies of bacterial longevity, and to ask the question, are there universal features of long-lived bacteria that we can uncover from studies of this proteobacterium? Several attributes make R. palustris a good model for addressing this question. First, it generates ATP and proton motive force from light, even when it is not growing (Fig. 1). Also, R. palustris carries out photophosphorylation under anaerobic conditions and does not generate oxygen, thus avoiding complications of oxidative stress during growth arrest. A transposon sequencing (Tn-seq) study of growth-arrested R. palustris identified 116 genes, which we call longevity genes, that are essential for the long-term viability of nongrowing cells but are not required for growth (17). Among these are genes that are conserved in bacteria and associated with basic cellular processes, including protein synthesis. Here, we carried out a physiological characterization of R. palustris in its growth-arrested but viable state. We found that active translation and optimized ribosomes were critical for the longevity of R. palustris, as was guanosine polyphosphate [(p)ppGpp]. This goes against the conventional description of nongrowth as state of metabolic inactivity often referred to as dormancy. We suggest that protein synthesis and other cellular activities are likely important for many bacteria to maintain viability when they are not growing.

## RESULTS

### Conditions of growth arrest.

In this study, R. palustris was grown anaerobically in minimal salts medium in sealed glass tubes in light until growth arrest occurred due to carbon source depletion (17). Cultures were incubated post-growth arrest in the same tubes in which they were grown. Nitrogen was supplied in excess as ammonium, and the carbon supplied was 20 mM sodium acetate. We took as day 0 of growth arrest the time at which cells stopped growing, as measured by a leveling off of the increase in the optical density (OD) of the cultures. Cells grew to twice the OD when we supplied them with 40 mM acetate, confirming that growth arrest occurred due to carbon source limitation.

### Characterization of cell size.

**FIG 2:**
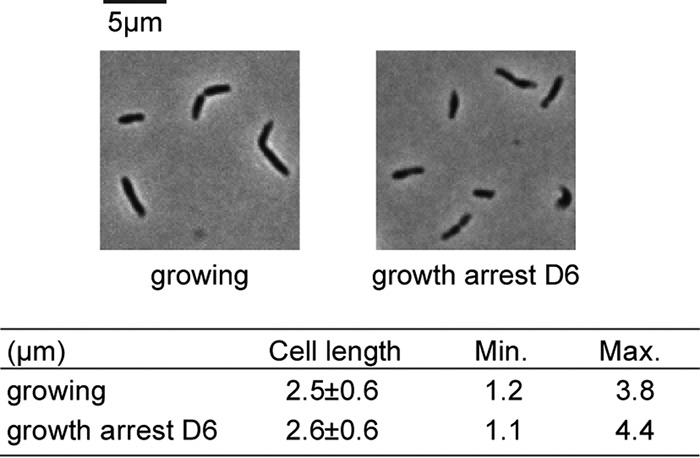
Comparison of lengths of R. palustris cells from growing cultures and from cultures at day 6 (D6) post-growth arrest. Cells were photographed at ×100 magnification with a Nikon Eclipse 80i digital microscope (*n* > 50). Min., minimum; Max., maximum.

Upon growth arrest due to starvation for nutrients, many bacteria decrease in size and change from a rod-shaped to a coccid form (6, 18–20). The degradation of ribosomes that accompanies growth arrest typically results in a loss of total cellular protein and RNA, which likely contributes to cell shrinkage. To put our work on R. palustris into context with the large body of work on starvation survival of heterotrophic bacteria, we measured cell size before and after growth arrest. As shown in Fig. 2, cells in log phase and at 6 days post-growth arrest do not differ appreciably in length or overall morphology.

### Growth-arrested R. palustris cells do not appear to undergo cycles of death and regrowth.

**FIG 3:**
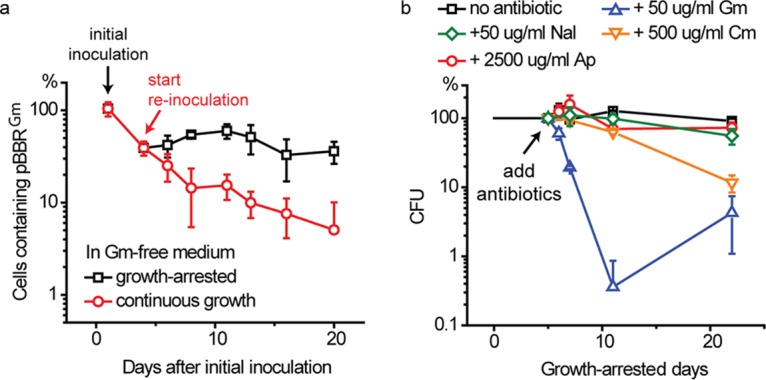
Growth-arrested R. palustris cells do not appear to undergo cycles of death and regrowth. (a) Percentage of R. palustris cells that continue to maintain plasmid pBBR^Gm^ during continuous growth (reinoculated in medium with fresh carbon source repeatedly) or during growth arrest (no treatment after the initial carbon source was depleted). Gm-free medium was used for this experiment. (b) Growth-arrested R. palustris is sensitive to antibiotics targeting ribosomes. Antibiotics were added 5 days after growth arrest to independent cultures. Gm, gentamicin; Cm, chloramphenicol; Nal, nalidixic acid; Ap, ampicillin. Error bars indicate the standard deviation (*n* > 3).

To put the work reported here in context with another aspect of the literature on starvation, we note that depending on the conditions of growth arrest, some bacteria, the best studied of which is Escherichia coli, undergo cycles of death and growth after entry into stationary phase (1, 21). Typically, a fraction of cells survive long-term stationary phase by feeding on nutrients released from dead cells. This leads to heterogeneity among cells, making it difficult to carry out meaningful studies of growth-arrested bacteria at the population level. Also, genetic heterogeneity develops in populations of growth-arrested bacteria, as there is strong selection pressure for mutations that enable cannibalism (22, 23). In previous work, we reported that populations of growth-arrested R. palustris cells were nearly 100% viable (17). To firmly establish that cells were truly growth arrested, rather than undergoing cycles of growth and death, we introduced an unstable plasmid expressing a gentamicin (Gm) resistance gene into wild-type R. palustris. When Gm is absent, the percentage of growing cells that retain this plasmid would be expected to decrease (24). On the other hand, a nongrowing culture would be expected to retain the plasmid in Gm-free medium. When this strain (WT::pBBR^Gm^) was inoculated into Gm-free medium, about 60% of the cells had lost the plasmid by the time they reached stationary phase. When these cells were transferred into fresh Gm-free medium and successively transferred several times over a period of 20 days after reaching stationary phase, the plasmid continued to be lost. In contrast, growth-arrested cells retained the plasmid over the same period of time (Fig. 3a). This suggests that most or all of the cells were indeed in a nongrowing state. However, we cannot exclude that some small percentage of the cells underwent cycles of growth and death.

In further support of the conclusion that growth-arrested R. palustris does not undergo major cycles of growth and death, we found that nongrowing cells were tolerant to the cell wall synthesis inhibitor ampicillin and the DNA synthesis inhibitor nalidixic acid at concentrations that prevented growth (Fig. 3b; see also Fig. S1 in the supplemental material). However, we also found that the viabilities of nongrowing cells were compromised by protein synthesis inhibitors (Fig. 3b and S1).

10.1128/mBio.02189-19.1 FIG S1 Responses of R. palustris to various antibiotics at different growth stages. Antibiotics are added to growing cultures or five days after growth arrest. Cm, chloramphenicol; Nal, nalidixic acid. Error bars indicate the standard deviation (*n* > 3). For growing cells treated with Nal, the number of viable cells was determined by *A*_660_, and a representative result is shown (*n* > 3). Download FIG S1, PPT file, 0.1 MB. Copyright © 2019 Yin et al. 2019 Yin et al. This content is distributed under the terms of the Creative Commons Attribution 4.0 International license.

### Long-lived, growth-arrested cells continue to synthesize proteins.

Growth-arrested R. palustris cells were sensitive to Gm, an aminoglycoside antibiotic that affects protein synthesis by causing mistranslation, which results in an accumulation of damaged proteins. We found that over 99% of growth-arrested cells lost viability over a few days when treated with Gm at a final concentration of 50 μg/ml (Fig. 3b). Although cells died relatively rapidly, they were not as sensitive to Gm as growing cells, which died within a few hours after exposure to this antibiotic. We noticed that the viability of growth-arrested cells exposed to Gm at 20 days post-growth arrest tend to be higher than the number of viable cells at day 10 post-growth arrest (Fig. 3b). It is possible that some cells developed resistance to Gm or that the Gm supplied may have become degraded, allowing for a small amount of growth on debris released from dying cells. Chloramphenicol (Cm) belongs to another class of antibiotic that targets ribosomes. Although typically bacteriostatic, Cm was effective in killing growth-arrested R. palustris over a prolonged period of time (Fig. 3b and S1). These results suggest that active translation is important for R. palustris longevity.

**FIG 4:**
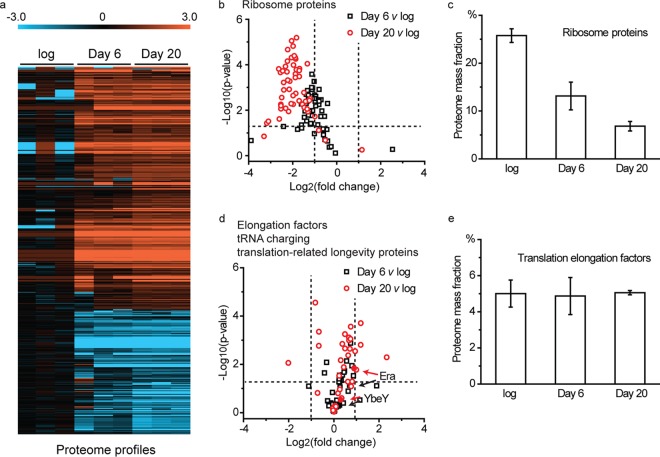
R. palustris protein abundances. Growing cells and growth-arrested cells are compared. (a) Log_2_ fold changes in the abundances of differentially abundant (*P* ≤ 0.05 by Student's *t* test) R. palustris proteins in growing cells (log), 6 days after growth arrest (day 6), and 20 days after growth arrest (day 20). The proteome profiles of three samples (*n* = 3) for each condition are shown (Table S1). (b) The average relative quantities of 53 identified ribosome proteins at day 6 (black, day 6) and day 20 (red, day 20) growth arrest relative to the growing cells (log). The horizontal dashed line indicates the position of *P* = 0.05 (Student's *t* test), and the vertical dashed lines indicate the position of 2-fold abundance changes. (c) Ribosome proteins compared in growing cells (log) and at day 6 and day 20 post-growth arrest as the % mass faction of the proteome (Table S3). Error bars represent the standard deviation. (d) The average relative quantities of selected proteins at day 6 (black, day 6) and day 20 (red, day 20) growth arrest relative to the growing cells (log). The horizontal dashed line indicates the position of *P* value of 0.05 (Student's *t* test), and the vertical dashed lines indicate the position of 2-fold abundance changes. This panel includes proteins involved in tRNA charging (KEGG map00970, Table S2), translation elongation factors (Table S2), and two previously identified longevity proteins (YbeY and Era). (e) Translation elongation factors (Table S3) compared in growing cells (log) and at day 6 and day 20 post-growth arrest as the % mass fraction of the proteome. Error bars represent the standard deviation. v, versus.

Proteomics experiments revealed that after 6 days and 20 days of growth arrest, 289 and 525 proteins, respectively, were present at >2-fold-higher levels than in growing cells (Fig. 4a and Table S1). Among these were enzymes involved in the degradation of compounds, including benzoate and fatty acids (Tables S1 and S2), that are preferred carbon sources for R. palustris, suggesting that cells respond to growth arrest by increasing the translation of proteins that may aid in scavenging carbon. In the period between 6 and 20 days post-growth arrest, 51 proteins of unknown function increased in abundance. Some proteins (163 and 310 proteins at days 6 and 20 post-growth arrest, respectively) were diminished in abundance by 2-fold or more following growth arrest (Fig. 4a and Table S1). This set included 53 out of 55 ribosomal proteins that were identified in our proteomics data set. On average, ribosome proteins were at approximately 50% and 25% of their original levels after 6 days and 20 days of growth arrest (Fig. 4b and c). We calculated the relative mass of these proteins per cell and from this calculated that ribosomes comprised 25.7% of the total protein mass in growing cells, which was reduced to 13.1% (49% reduction) and 6.8% (74% reduction) after 6 and 20 days of growth arrest, respectively (Fig. 4c and Table S3). Not all translation-related proteins were decreased in abundance. For example, translation elongation factors made up ∼5% of the total protein from growing cells as well as growth-arrested cells (Fig. 4d and e and Table S3). Similar patterns were also observed for proteins involved in aminoacyl-tRNA biosynthesis (tRNA charging) and for two previously identified longevity proteins YbeY and Era (17) that are involved in ribosome maturation (Fig. 4d).

10.1128/mBio.02189-19.5 TABLE S1 Proteome of R. palustris in log phase and at 6 days and 20 days post-growth arrest. Download Table S1, XLSX file, 0.5 MB. Copyright © 2019 Yin et al. 2019 Yin et al. This content is distributed under the terms of the Creative Commons Attribution 4.0 International license.

10.1128/mBio.02189-19.6 TABLE S2 Categories (KEGG) of proteins with changes in abundances in growth-arrested compared to growing R. palustris cells. Download Table S2, XLSX file, 0.1 MB. Copyright © 2019 Yin et al. 2019 Yin et al. This content is distributed under the terms of the Creative Commons Attribution 4.0 International license.

10.1128/mBio.02189-19.7 TABLE S3 Relative mass % of individual R. palustris proteins compared to the whole-cell proteome in log-phase cells and in cells at 6 days and 20 days post-growth arrest. Download Table S3, XLSX file, 0.5 MB. Copyright © 2019 Yin et al. 2019 Yin et al. This content is distributed under the terms of the Creative Commons Attribution 4.0 International license.

Finally, to directly examine protein synthesis in growth-arrested R. palustris, we constructed a LacZ reporter driven by a P_hirI_ promoter that can be induced with phenylacetyl-homoserine lactone (PA-HSL) (Fig. S2). After 6 days of growth arrest, PA-HSL was added to the culture, and samples were collected after 2 additional days of incubation. The negative control without added signal showed a base level of LacZ expression. Compared to this, LacZ activity was ∼70% higher when the PA-HSL signal was added to induce *lacZ* expression (Fig. S2). This observation directly demonstrates that R. palustris has the ability to synthesize proteins during growth arrest.

10.1128/mBio.02189-19.2 FIG S2 The *hirR* gene is expressed with a glucose dehydrogenase (GDH) (*rpa0944*) promoter, and the LacZ protein is expressed with the P_hirI_ promoter. An R. palustris culture was grown to day 6 of growth arrest, and the PA-HSL signal was added into the culture. After two days, the samples were collected and the level of LacZ was analyzed as stated in Materials and Methods. With the signal added, the LacZ level was ∼70% higher than in the negative control. Download FIG S2, PPT file, 0.1 MB. Copyright © 2019 Yin et al. 2019 Yin et al. This content is distributed under the terms of the Creative Commons Attribution 4.0 International license.

### Characterization of protein synthesis apparatus in long-lived cells.

**FIG 5:**
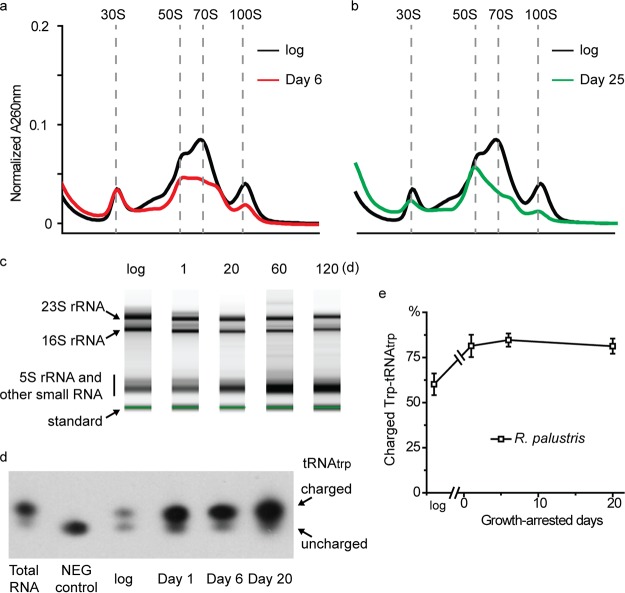
Translation is intact and active in growth-arrested R. palustris. (a) The ribosome profile of cells at 6 days post-growth arrest is shown and compared to ribosomes from growing R. palustris (log). (b) The ribosome profile of cells at 25 days post-growth arrest is shown and compared to that of ribosomes from growing R. palustris cells (log). (c) Total RNA was purified and analyzed from growing and growth-arrested R. palustris cells. The same amount of RNA was loaded in each lane. The quantification of replicated experiments is shown in Fig. S3. The standard is the internal standard of the RNA ScreenTape system used (Agilent). (d) The charged and uncharged species of tRNA_trp_ are shown here with a representative Northern blot. The aminoacylated (charged) and deacylated (uncharged) species of tRNA_trp_ were separated with an acid urea gel and probed with a tRNA_trp_ probe. NEG, negative. (e) Quantification of the charged and uncharged species of tRNA_trp_ as analyzed in Fig. 5d. Error bars represent the standard deviation (*n* > 3).

We isolated R. palustris ribosomes by sucrose gradient centrifugation and found that the ribosome profile of growing R. palustris cells included 30S, 50S, and 70S populations, as is typical for growing bacteria (Fig. 5a). There was also a small peak corresponding to a 100S population, which is likely a dormant form of ribosomes, as has been seen in other bacteria (25, 26). The ribosome profile of growth-arrested R. palustris cells resembled that of growing cells (Fig. 5a and b), but the quantity of the 70S population was lower at day 6 and even lower at day 25 post-growth arrest, suggesting that cells degraded some fraction of their ribosomes after they stopped growing. This is consistent with our proteomics data (Fig. 4b) and fits the notion that the total rate of protein synthesis is expected to be much lower in growth-arrested cells than in growing cells. Ribosome degradation is often accompanied by the nonspecific degradation of rRNA (27–30). However, even after 120 days of growth arrest, cells retained intact species of 23S and 16S rRNA, indicating that cells continuously repaired and replaced ribosomes albeit at probably a low rate after they stopped growing (Fig. 5c and S3). The relative levels of aminoacylated (or charged) and free forms of tRNAs are important for promoting and inhibiting translation (31). We used Northern blot analysis to estimate the percentage of tryptophan-charged tRNA_trp_ (Trp-tRNA_trp_) relative to tRNA_trp_. In growing cells, ∼60% of tRNA_trp_ was in the charged Trp-tRNA_trp_ form (Fig. 5d and e). This level is comparable to that reported in other bacteria (32). The percentage of Trp-tRNA_trp_ increased to ∼85% upon growth arrest and was maintained at this level for ∼20 days (Fig. 5d and e).

10.1128/mBio.02189-19.3 FIG S3 rRNA levels in different R. palustris strains at different growth stages. The quantities of 16S, 17S, and 23S rRNA are determined using an RNA ScreenTape system (Agilent) and normalized against the levels of 23S rRNA in growing cells of wild-type R. palustris. An unpaired *t* test was performed with at least three replicates (n.s., not significant; *, *P* < 0.05; **, *P* < 0.01; ***, *P* < 0.005; ****, *P* < 0.001, as determined by Student’s *t* test). Download FIG S3, PPT file, 0.1 MB. Copyright © 2019 Yin et al. 2019 Yin et al. This content is distributed under the terms of the Creative Commons Attribution 4.0 International license.

### Two proteins identified as essential for R. palustris longevity are critical for ribosome integrity in growth-arrested cells.

**FIG 6:**
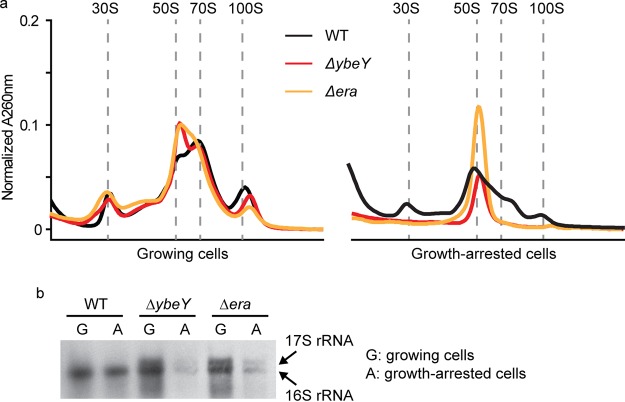
YbeY and Era are important for ribosome maintenance during growth arrest. (a) Deletion of either *ybeY* or *era* had little effect on ribosomes from growing R. palustris cells, while the ribosomes from growth-arrested cultures of *ybeY* and *era* deletion mutants lacked 30S subunits. (b) R. palustris total RNA was analyzed by Northern blotting with a probe targeting 16S rRNA. In growing cells, both the Δ*ybeY* and Δ*era* mutants accumulated 17S rRNA but maintained substantial levels of 16S rRNA. In growth-arrested cells, both 17S rRNA and 16S rRNA species were depleted.

Among the longevity genes that we have identified in R. palustris (17), *ybeY* and *era* are predicted to encode proteins that interact with each other and the S11 ribosome protein during ribosome maturation (33–37). *ybeY* or *era* deletion mutants have compromised longevity but grow normally (17). Here, we found that the ribosome profiles of growing cells were similar between the wild type and these mutants (Fig. 6a). All had distinctive 30S, 50S, and 70S populations, although the 50S subunit was slightly more abundant in the mutants. The ribosome profiles of growth-arrested Δ*ybeY* and Δ*era* mutant cells were noticeably different from that of the wild type, however. A prominent 50S subunit peak was seen, but the amounts of 30S subunits and 70S ribosomes were greatly diminished (Fig. 6a).

As predicted based on studies of their E. coli homologs (33–36), Δ*ybeY* and Δ*era* mutant strains were defective in 16S rRNA maturation. An extra band immediately above the 16S rRNA was present in gels of RNA from both mutants (Fig. S3). Northern blot experiments confirmed that the extra band was 17S rRNA, a precursor of 16S rRNA (Fig. 6b). In growth-arrested cells, both 16S rRNA and 17S rRNA species were severely depleted in the mutated strains (Fig. 6b and S3). This is likely connected to the defective 30S ribosome subunit production shown in Fig. 6a, as well as the compromised longevity of R. palustris. We do not know why the 17S and 16S rRNAs were so much lower in abundance in growth-arrested versus growing cells (Fig. 6b and S3). It is possible that factors associated with these mutations coupled with the stress of growth arrest lead to their degradation. *ybeY* or *era* deletion mutants had wild-type levels of charged tRNA-Trp (Fig. S4), suggesting that tRNA aminoacylation is not directly coordinated with the process of ribosome maturation.

10.1128/mBio.02189-19.4 FIG S4 Trp-tRNATrp remained highly charged in the growth-arrested cells of the Δ*ybeY* and Δ*era* mutant strains. Download FIG S4, PPT file, 0.8 MB. Copyright © 2019 Yin et al. 2019 Yin et al. This content is distributed under the terms of the Creative Commons Attribution 4.0 International license.

**FIG 7:**
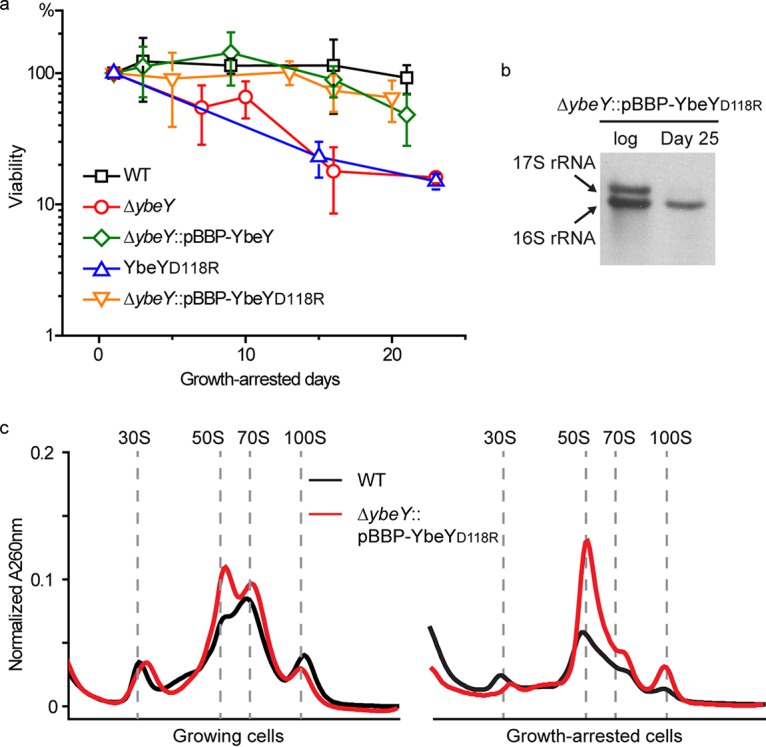
Overexpression of YbeY_D118R_ complemented the longevity defect of a Δ*ybeY* mutant. (a) Longevity of different R. palustris strains. Deletion of the *ybeY* gene compromised the longevity of growth-arrested cells (Δ*ybeY* mutant), and this was complemented by expressing this gene in *trans* (Δ*ybeY*::pBBP-YbeY mutant) (17). A *ybeY*_D118R_ mutant had a longevity defect similar to that of the Δ*ybeY* mutant. Overexpression of *ybeY*_D118R_ in *trans* restored the longevity of a Δ*ybeY* mutant (Δ*ybeY*::pBBP-YbeY_D118R_). Error bars indicate the standard deviation (*n* > 3). (b) R. palustris total RNA was analyzed by Northern blotting with a probe targeting 16S rRNA. In the growth-arrested cells of the Δ*ybeY*::pBBP-*ybeY*_D118R_ mutant, only 16S rRNA appeared, and no 17S rRNA was detected. (c) The ribosomes from the Δ*ybeY*::pBBP-YbeY_D118R_ mutant were analyzed on a sucrose gradient. In the growth-arrested cells, 30S, 50S, and 70S ribosomes were clearly seen, although the abundance of the 50S subunit is higher than that of the wild type (WT).

As shown in Fig. 4b, there was no significant change in the abundances of YbeY and Era proteins in growth-arrested cells. Since the core ribosomal proteins were decreased 2- to 4-fold in abundance (Fig. 4b), the relative abundances of YbeY and Era were increased on a per-ribosome basis following growth arrest. To test whether we could change the effects of YbeY on longevity by modulating its relative abundance, we constructed and expressed a YbeY variant predicted based on work in E. coli to interact weakly with ribosomes. In E. coli, the interaction between YbeY and ribosomes is partially disrupted by replacement of a conserved aspartate with an arginine (37). When we made the corresponding YbeY_D118R_ change in R. palustris, the strain was compromised in its longevity to a similar degree as the Δ*ybeY* mutant (Fig. 7a). When we overexpressed YbeY_D118R_ in *trans* (Δ*ybeY*::pBBP-*ybeY*_D118R_), however, the longevity defect was complemented (Fig. 7a). The quantity of 16S rRNA was similar to that of the wild type, and no 17S rRNA was observed during growth arrest (Fig. 7b). The ribosome profile of the growth-arrested Δ*ybeY*::pBBP-*ybeY*_D118R_-complemented strain differed from that of growth-arrested wild-type cells, especially in the quantities of 50S and 70S ribosomes (Fig. 7c). However, growth-arrested cells could apparently tolerate these differences when challenged in longevity assays (Fig. 7a). These results suggest that it may be helpful for growth-arrested cells to have relatively more YbeY protein than growing cells and that this promotes 30S ribosome subunit assembly under these conditions.

### The stringent response is important for longevity.

**FIG 8:**
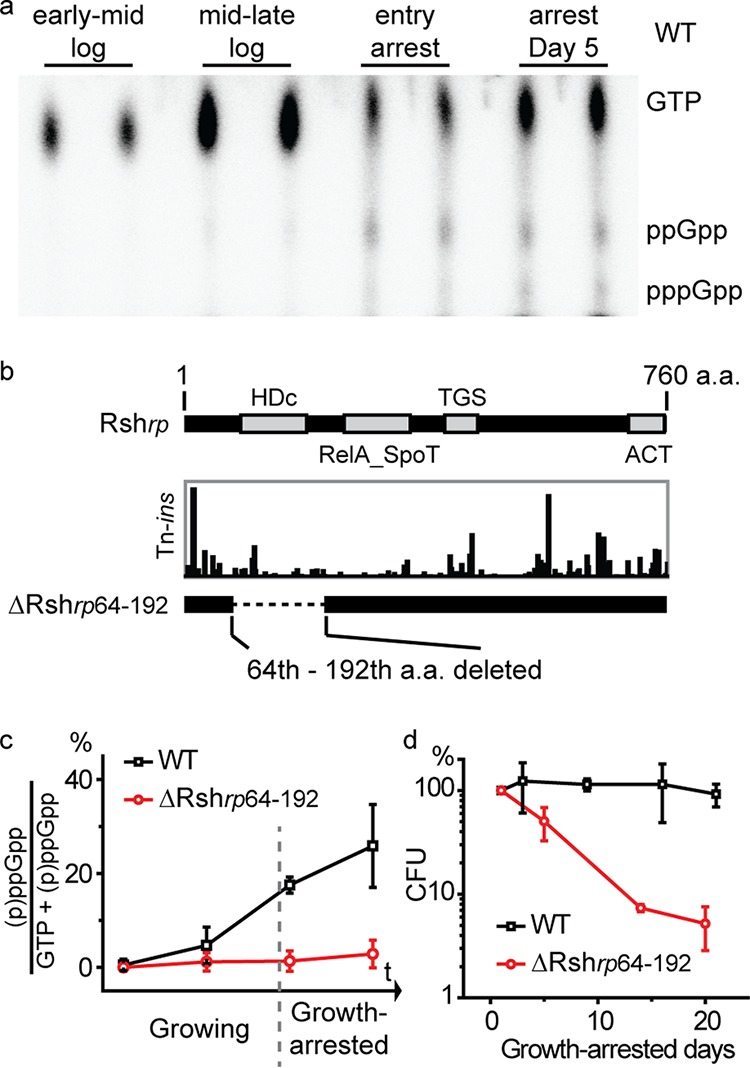
Growth-arrested cells with very low levels of (p)ppGpp have compromised longevity. (a) The level of (p)ppGpp was low in exponentially growing R. palustris cells and increased during growth arrest. (b) Domain structure of Rsh*_rp_* (top). Locations and relative quantities of Tn insertions in Rsh*_rp_* are shown (17). A ΔRsh*_rp_*_64–192_ mutant was made by deleting the sequence encoding amino acids (a.a.) 64 to 192 of Rsh*_rp_* from R. palustris. (c) (p)ppGpp synthesis was severely compromised in the ΔRsh*_rp_*_64–192_ mutant strain. The time points shown correspond to the time points shown in panel A, representing early mid-log-phase, mid-late-log phase, entry into growth arrest, and day 5 following growth arrest. (d) The longevity of the Δ*rsh_rp_*_64–192_ mutant was severely compromised. Less than 10% of the cells remained viable after 2 weeks of growth arrest. Error bars indicate the standard deviation (*n* > 3).

Our results suggest that translation is reduced but still maintained in growth-arrested cells. A well-known mechanism that contributes to this is the stringent response mediated by guanosine polyphosphate [(p)ppGpp] (38, 39). In many bacteria, the level of (p)ppGpp increases upon growth arrest, and this inhibits the transcription of ribosomal genes, although the mechanism by which this is achieved varies among bacteria (38–40). As with other bacteria, we detected little to no (p)ppGpp in exponentially growing R. palustris cells. However, the level of (p)ppGpp increased sharply as growing cells reached stationary phase and remained at relatively high levels after growth arrest (Fig. 8a).

Unlike E. coli, but like most other bacteria outside the *Gammaproteobacteria* and *Betaproteobacteria*, R. palustris has a single bifunctional (p)ppGpp synthesis/hydrolysis *rsh* gene, here called *rsh_rp_*, that has multiple domains, including a hydrolase (HDc) domain and a RelA_SpoT domain (Fig. 8b). Our Tn-seq results indicated that this gene (*rpa2693*) was essential for R. palustris longevity but not for growth (17, 41, 42). However, we were unable to delete the full-length *rsh_rp_* gene. A subsequent inspection of the locations of the Tn insertions in this gene in our bank of Tn-mutagenized cells revealed that there were relatively few insertions in its RelA_SpoT domain (Fig. 8b). When we made domain specific deletions in Rsh*_rp_*, we obtained a hydrolase domain deletion mutant (ΔRsh*_rp_* from amino acids 64 to 192 [ΔRsh*_rp_*_64–192_]; Fig. 8b) that produced very little (p)ppGpp during growth or growth arrest (Fig. 8c). We do not know why this deletion affected (p)ppGpp synthesis in this way, but one idea is that it destabilized or altered the structure of the protein so that its ability to synthesize (p)ppGpp was compromised. The ΔRsh*_rp_*_64–192_ mutant grew with a generation time of 9.4 ± 0.6 h, compared to a wild-type generation time of 6.4 ± 0.2 h, which is a growth rate difference of about 30%. In contrast to this relatively modest effect on the growth rate, the ΔRsh_rp64-192_ mutation had a severe effect on longevity. As shown in Fig. 8d, the mutant survived poorly in growth arrest.

## DISCUSSION

We found that illuminated R. palustris responds to growth arrest by reducing ribosome abundance, as is typical of most bacteria when their growth rate decreases. Growth-arrested cells continued to synthesize proteins, some at elevated levels relative to growing cells, over the period of 20 days that we followed protein composition by proteomics (Table S1), and it is likely that they repaired and replaced some portion of their original ribosome inventory over this time period. The levels of most proteins involved in tRNA charging and translation elongation and were at similar levels in growing and growth-arrested cells (Fig. 4d and Table S2), which is consistent with the notion from a previous report that elongation rates are maintained as bacterial growth rates decrease even toward zero growth (43). In addition, a tRNA that we followed was almost fully charged 20 days into growth arrest (Fig. 5d and e). Not only does protein synthesis continue after growth arrest, but it is essential for the viability of nongrowing cells, as indicated by their sensitivity to Gm and Cm (Fig. 3b and S1).

There are reports of continued protein synthesis in other species of bacteria in growth arrest, the more convincing of which come from studies in which the production of fluorescent proteins in single cells was used as a proxy for protein synthesis. Antibiotic persisters in early stages of formation, as well as nongrowing E. coli, Mycobacterium sp., and Salmonella sp. cells, have all been inferred to carry out protein synthesis (44–47). These results suggest that maintenance of some level of protein synthesis may be required for the viability of many nongrowing bacteria. From this, it follows that it would make sense to prioritize the development of antibiotics that target protein synthesis in growth-arrested cells. This will likely require understanding in detail what happens to bacterial ribosomes following growth arrest. Bacterial ribosomes are known to be differentially posttranslationally modified in stationary-phase cells (48). Two longevity genes, *ybeY* and *era*, were essential for normal ribosomes to form in nongrowing R. palustris cells (Fig. 6a), and their encoded proteins increased in abundance on a per-ribosome basis during growth arrest (Fig. 4b). Era is a well-studied bacterial GTPase (36), and genes for it and YbeY are present and conserved in most bacteria (34). Our data are consistent with those from studies of the corresponding E. coli proteins showing that YbeY and Era are involved in rRNA processing in R. palustris. When we looked at rRNAs formed by the Δ*ybeY* and Δ*era* mutants that were actively growing, it was apparent that growing cells and growth-arrested cells had the same defect in 17S rRNA processing (Fig. 6b), but only the growth-arrested cells had a physiological phenotype. This highlights a feature of at least some R. palustris longevity proteins, which is that they are functional in growing cells as well as in nongrowing cells, but it is only when the additional demands of growth arrest on cellular functionality are imposed that essentiality becomes apparent in growth arrest. Our work on Era and YbeY has led us to conclude that optimized ribosomes are a feature of long-lived cells.

In early stationary phase and when in growth arrest, R. palustris synthesizes increasing amounts of (p)ppGpp, indicating that the cells mount a stringent response. The gene *rpa2693*, which we call *rsh_rp_*, is among the genes identified by Tn-seq to be essential for longevity (17). A Δ*rsh*_rp64-192_ mutant did not produce detectable (p)ppGpp, and the viability of this mutant decreased rapidly after growth arrest (Fig. 8c and d). There is good evidence that (p)ppGpp is also important for the development of antibiotic persisters (49) and for long-term survival of latent and growth-arrested M. tuberculosis cells (50, 51). A great deal still needs to be learned about how (p)ppGpp signaling is activated in growth-arrested R. palustris cells. Studies of (p)ppGpp in different species of bacteria have generally not followed the levels of this nucleotide deep into stationary phase. E. coli has been shown to have elevated levels of (p)ppGpp only in early stationary phase, with (p)ppGpp levels returning to baseline later in stationary phase. This is very different from the situation in R. palustris, where high levels of (p)ppGpp are maintained for at least 5 days post-growth arrest. Uncharged tRNAs are understood to be the classic signal for activation of RelA and upregulation of (p)ppGpp synthesis in E. coli and other gammaproteobacteria (40). However, in R. palustris, tRNA_Trp_ charging is even higher in growth arrest than during growth (Fig. 5d and e). It could be that one or several tRNA species other than tRNA_Trp_ become uncharged during carbon starvation, which could potentially trigger (p)ppGpp synthesis in a fashion similar to the case of E. coli. However, it is also known that the exact triggers of (p)ppGpp synthesis can vary between species (40, 52, 53). For example, amino acid starvation that typically leads to uncharged tRNA species is insufficient to trigger (p)ppGpp accumulation in Rhizobium meliloti (54, 55), Caulobacter crescentus (56), and Rhodobacter sphaeroides (57). A nitrogen-related phosphotransferase system (PTS^NTR^) has been found to be important for stimulating (p)ppGpp production in C. crescentus in response to nitrogen limitation (58, 59), but the mechanism by which carbon limitation, the condition studied here, may stimulate (p)ppGpp accumulation in alphaproteobacteria remains unclear (56).

Other future areas of investigation are to understand why (p)ppGpp is essential for R. palustris longevity and to identify its target genes and proteins. Based on work with other bacteria, it is very likely that (p)ppGpp interacts with RNA polymerase to inhibit the transcription of genes encoding ribosomal RNAs and proteins (38–40). However, this would need to be done in a very controlled fashion because some new ribosome biogenesis is very likely to be required for R. palustris longevity in growth arrest. (p)ppGpp has recently been shown to bind to Era from Staphylococcus aureus and E. coli to inhibit its GTPase activity (60–62), and a similar effect on Era from R. palustris might influence the biogenesis and integrity of its ribosomes.

Virtually all bacteria probably stop growing at one time or another due to nutrient limitation in their native niches, and it is also likely that that they employ diverse strategies to stay alive. However, there also may be evolutionarily conserved core survival strategies that all bacteria use. We suggest that optimized ribosomes, a stringent response, and protein synthesis are part of this core strategy. The primary reason for the extreme longevity of R. palustris is likely its ability to continuously generate ATP by photophosphorylation when it is in growth arrest, because growth-arrested cells incubated in the dark do not survive nearly as well (17). All bacteria need ATP to stay alive. There is plenty of evidence in the literature suggesting that ATP obtained by scavenging resources from the environment or from the degradation of internal energy stores or cell biomass is necessary to support the viability of diverse non-spore-forming growth-arrested bacteria (20). A recent study also suggested that various ATP levels per cell might underlie the heterogeneity in populations and lead to nongrowing persisters (63). Protein synthesis and (p)ppGpp synthesis are core features that underlie the extraordinary longevity of R. palustris. Both of these processes are highly energy consuming and crucial for all bacteria. To understand how R. palustris integrates these processes and utilizes its ATP during growth arrest could be a key to understanding how nongrowing bacteria in general stay alive.

## MATERIALS AND METHODS

### Bacterial strains and growth and incubation conditions.

R. palustris strain CGA009 was used as the wild-type strain for this study. Anaerobic cultures were grown under illumination at 30°C, using a defined medium, PM (64), with 20 mM sodium acetate or 10 mM sodium succinate as the carbon source. The longevity of R. palustris cultures was determined by counting CFU, as described previously (17). In-frame deletions of R. palustris genes were created by using the suicide vector pJQ200SK, as described before (65). For in *trans* expression in R. palustris, the target genes were cloned into plasmid pBBPgdh, and the plasmids were mobilized into R. palustris by conjugation using E. coli strain S17-1 (66). Media were supplemented with 100 μg/ml gentamicin for in *trans* complementation experiments.

### Construction of inducible LacZ reporter and LacZ assays.

To construct the inducible LacZ reporter, we assembled a *lacZ* fragment and a *hirR*-P_hirI_ fragment (67) into a pBBP plasmid backbone (66) (Fig. S2). The promoter P_hirI_ responds specifically to PA-HSL signal (67). At day 6 of R. palustris growth arrest, PA-HSL was added to the culture at a final concentration of 1 μM. After an additional ∼48 h of incubation, 5 ml of culture was collected and resuspended in 2 ml of Z-buffer (60 mM Na_2_HPO_4_, 40 mM NaH_2_PO_4_, 12.5 mM NaCl, 1 mM MgSO_4_, and 40 mM 2-mercaptoethanol [pH 7.0]). The samples were sonicated at a frequency of 20 to ∼40 kHz for 3 cycles (20 s of sonication and 20 s cooling on ice). Cell lysis was cleared by centrifugation at 4°C and 15,000 rpm for 15 min, and the supernatant was collected for subsequent β-galactosidase assays. To start the reaction, 200 μl supernatant was mixed with 40 μl of 4 mg/ml o-nitrophenyl-β-d-galactopyranoside (ONPG) at 30°C, and the *A*_420_ was monitored using a BioTek H1 plate reader. The level of LacZ was calculated as the *V*_max_ of the reaction normalized by the total protein concentration of the supernatant.

### Ribosome purification.

Ribosomes from R. palustris cells were isolated following a method adapted from a previous study (68). About 35 ml of culture was grown to the desired state and poured over a 10-ml ice sheet (fast-cool). The ice sheet melted within 10 min, and the cells were collected by centrifugation at 4,000 rpm for 15 min. The cell pellet was resuspended in 2 ml of buffer A (20 mM HEPES-KOH [pH 7.5], 30 mM NaCl, 8 mM MgCl_2_) with 1 μl/ml RNasin (Promega). Three hundred microliters of 0.1-mm zirconia/silica beads (BioSpec Products) was added to every milliliter of sample. The samples were lysed with a Mini-Beadbeater-24 (BioSpec Products) at 3,500 rpm for 1 min at 4°C and kept on ice for 1 min, and the cycle was repeated 4 more times. The microbeads and cell debris were removed by centrifugation at 15,000 rpm for 10 min. The lysates were further clarified by spinning at 30,000 × *g* in a Beckman TL-1000 ultracentrifuge for 30 min, followed by pelleting the ribosomes at 100,000 × *g* for 4 h. The pellet was resuspended with buffer A overnight on ice.

### Sucrose gradient analysis.

About five *A*_260_ units of ribosomes (less than 200 μl in volume) were applied on top of 11 ml of a sucrose gradient (7 to 47%). The samples were centrifuged through the gradient at 39,000 rpm for 4.5 h with a SW41 rotor and then were separated with a Brandel gradient pump. The sucrose gradient was made with buffer A.

### rRNA analysis.

Total RNA was purified from 3 to 5 ml of R. palustris culture using an miRNeasy minikit. The rRNAs were then analyzed with an RNA ScreenTape system (Agilent).

To probe 16S rRNA by Northern blotting, 1 μg of total RNA was heated at 70°C for 10 min and immediately chilled on ice. The samples were then separated on a 1% agarose gel with 1× Tris-borate-EDTA (TBE) buffer. After electrophoresis, RNA was transferred to an Amersham Hybond-XL membrane (GE Life Sciences) with a downward nucleic acid transfer system overnight using 20× SSC (1× SSC is 0.15 M NaCl plus 0.015 M sodium citrate) buffer. A probe (5′-TCGTGCCTCAGCGTCAGTAATGGCCCAGTGA) labeled with digoxigenin (DIG) was used to detect 16S rRNA, following the manufacturer’s instructions (Roche).

### Charging analysis of tRNA_Trp_.

Total RNA was isolated with the QIAzol lysis reagent, according to the manufacturer’s instructions (Qiagen). The dried RNA pellet was dissolved and stored in 10 mM sodium acetate (pH 4.5). A deacylated control sample was prepared by incubating 10 μg of total RNA with 1 mM EDTA and 100 mM Tris-HCl (pH 9.0) at 42°C for an hour (69). Northern blot and acid-urea gel electrophoresis were performed essentially as described previously (32, 69). Loading dye (0.01% xylene cyanol, 0.01% bromophenol blue, 8 M urea, 1 mM EDTA, and 10 mM sodium acetate [pH 5.0]) was mixed with sample at a 1:1 ratio. Samples were loaded on a 12% acid-urea gel of 20 cm and then were run at 15 W for 12 h at 4°C. Transfer of RNA to an Amersham Hybond-XL membrane (GE Life Sciences) was performed in 0.5× TBE buffer at 650 mA for 30 min 4°C. The aminoacylated and the deacylated tRNA_Trp_ were detected using a DIG-labeled probe (5′-GGTTTTGGAGACCGGTGCTCTACCAATTGAG), following the manufacturer’s instructions (DIG; Roche).

### Determination of cellular (p)ppGpp levels.

*In vivo* (p)ppGpp labeling and detection were performed as described previously, with some modifications (70). Anaerobic R. palustris cultures were grown in 1.5 ml PM medium with 100 μCi/ml of Na_2_H^32^PO_4_ (product no. NEX011001MC; PerkinElmer) added at the time of inoculation. At the desired time points, 200 to 300 μl of culture was collected and extracted with 8 M formic acid. The supernatants of the extracts were spotted on polyethyleneimine (PEI)-cellulose thin-layer chromatography (TLC) plates (Sigma-Aldrich) and separated with 1.5 M KH_2_PO_4_ (pH 3.4). The plates were then dried and visualized by phosphor imaging. The level of (p)ppGpp was calculated by the ratio (ppGpp + pppGpp)/(GTP + ppGpp + pppGpp).

### Proteomic analysis.

Biological triplicates of cells were lysed, digested with trypsin, and analyzed by liquid chromatography-tandem mass spectrometry (LC-MS/MS), as described previously (71). The collected data were analyzed using the MaxQuant software (v 1.5.5.1) (41, 72) for peptide/protein identification and quantification. Identification of peptides was done by searching against the R. palustris ATCC BAA-98 (strain CGA009) sequences from the UniProt Knowledgebase (downloaded on 30 August 2016). The search was performed first with a larger parent mass tolerance (20 ppm) to identify peptides and recalibrate peptide masses. After recalibration, the parent mass tolerance was set to 4.5 ppm for a second round of search. During the searches, oxidation of methionine was a variable modification, and trypsin digestion was required in both peptide termini. Identifications were filtered with a 1% false-discovery rate at peptide-spectrum match and protein levels. The function “matching between runs” was checked to detect peptide peaks in LC-MS/MS runs that they were not fragmented. Label-free quantification (LFQ) values were used for quantitative analysis. This method considers only the most reliably detected peptides and normalizes according to the total signal of the run. It is therefore ideal for comparing individual proteins in different samples (73). For absolute quantification, intensity-based absolute quantification (iBAQ) (74) measurements of individual proteins were sequentially normalized by the protein molecular mass and total mass in the sample, resulting in relative protein mass compared to the entire cell proteome. Proteins were considered differentially abundant with a *P* value of ≤0.05 by *t* test considering two tails and equal variance.

### Data availability.

The mass spectrometry data have been deposited to the ProteomeXchange Consortium via the PRIDE partner repository with the data set identifier PXD013729.
