# Supplementary figures and images for: Bacterial Longevity Requires Protein Synthesis and a Stringent Response
Source: mBio. 2019 Oct 15;10(5):e02189-19. doi: 10.1128/mBio.02189-19 (PMC6794480; doi:10.1128/mBio.02189-19)

## Slide 1
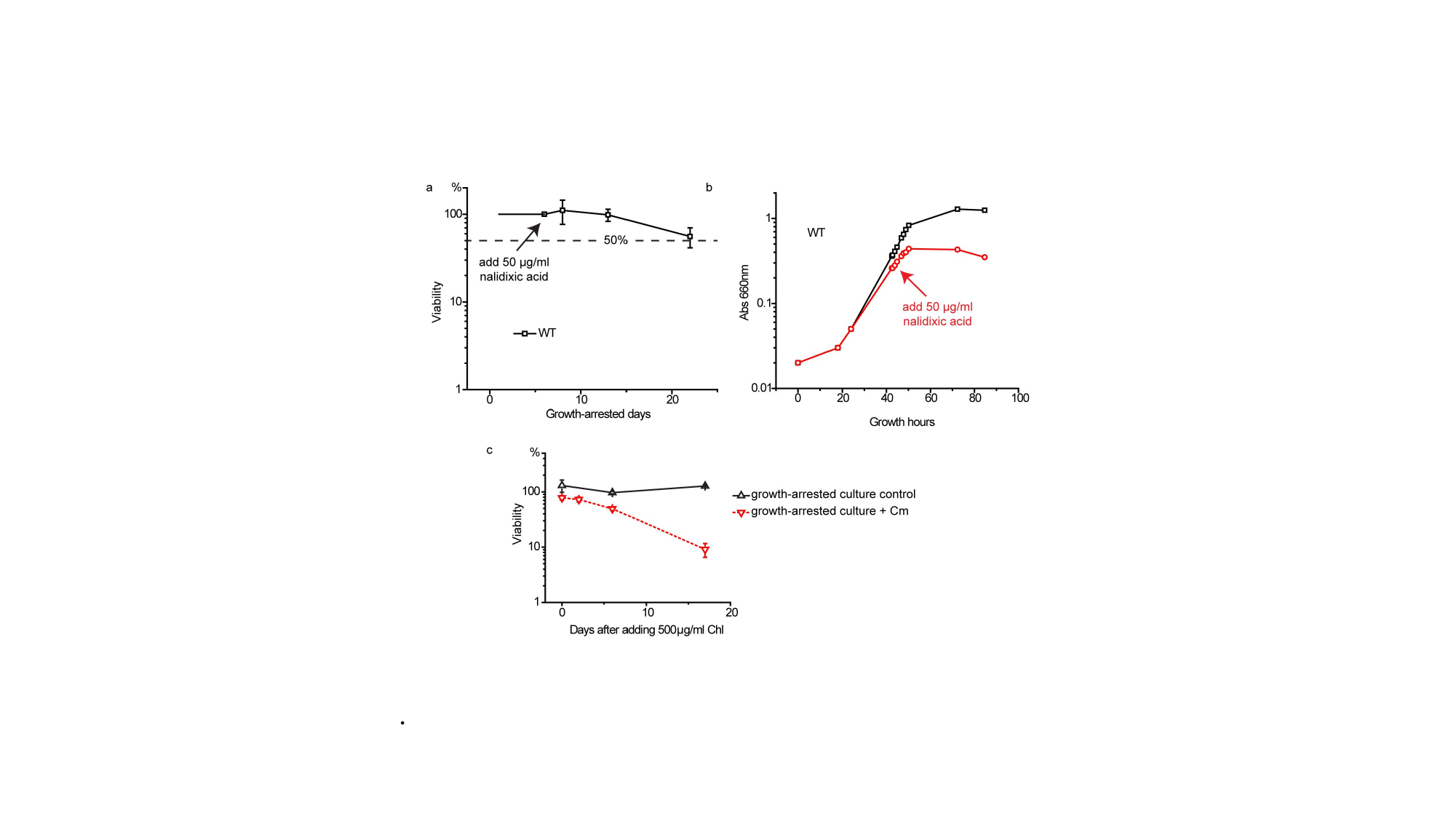

.

Supplement: FIG S1 [file mBio.02189-19-sf001.ppt]

## Slide 1
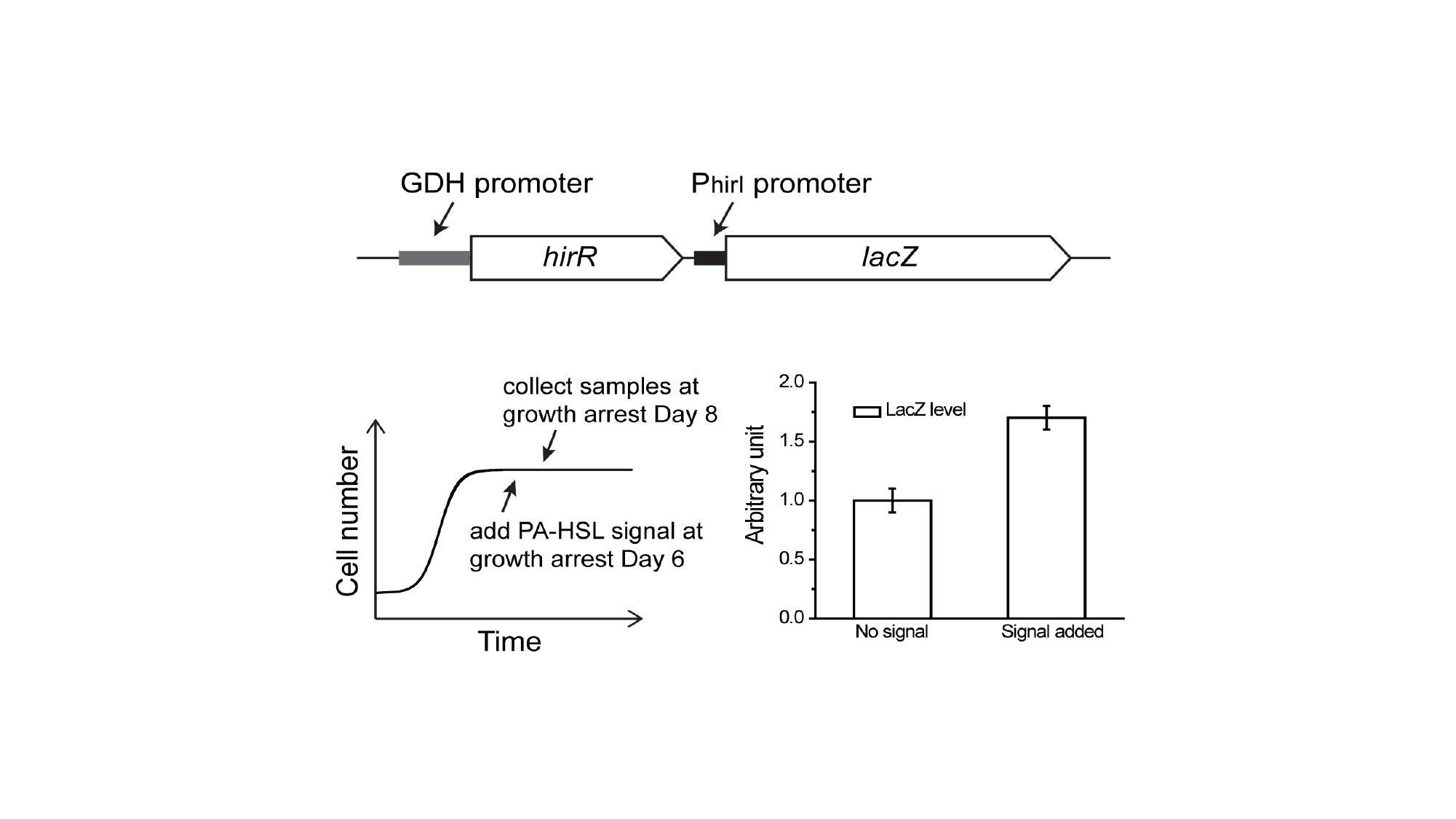

Supplement: FIG S2 [file mBio.02189-19-sf002.ppt]

## Slide 1
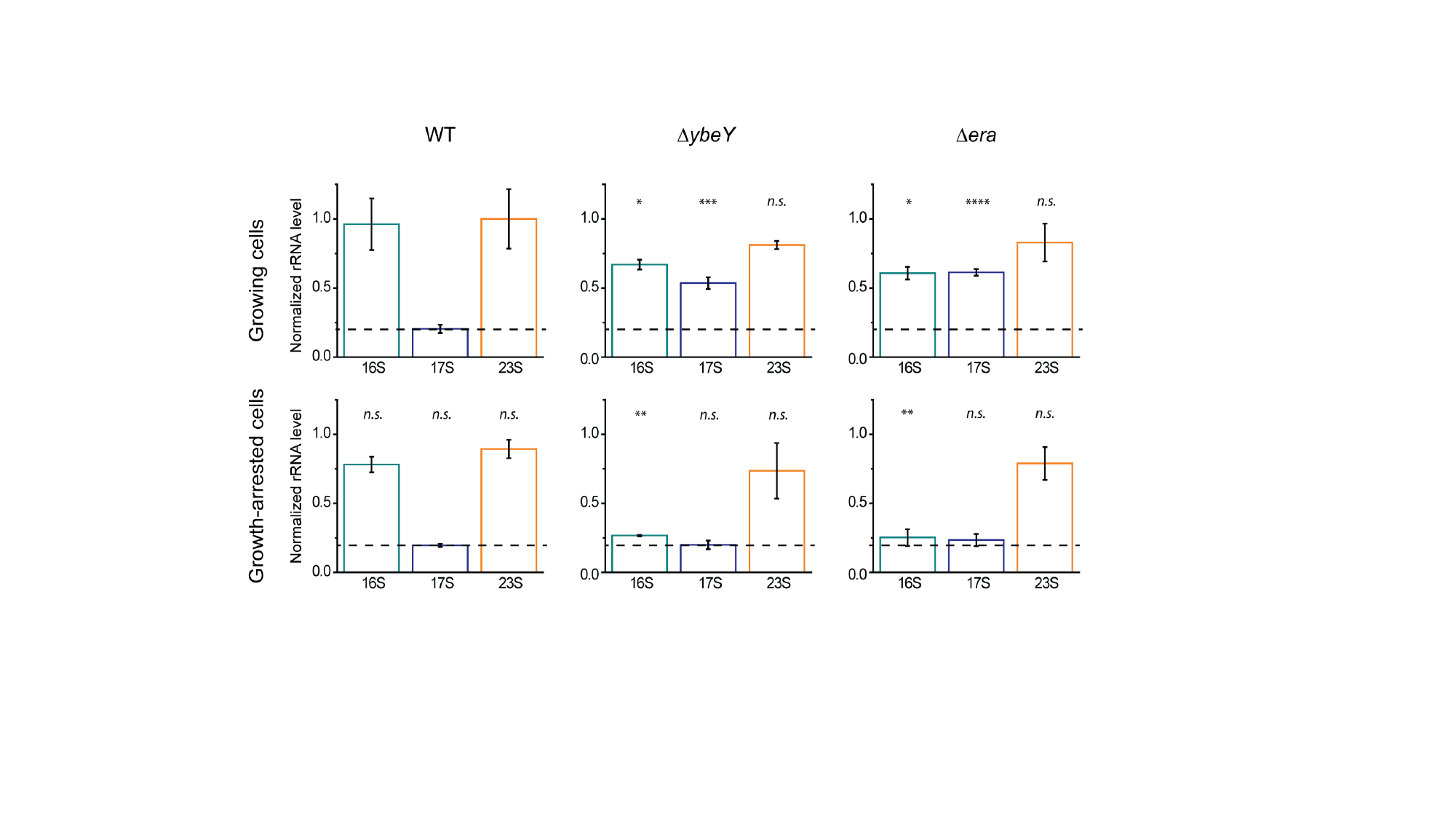

Supplement: FIG S3 [file mBio.02189-19-sf003.ppt]

## Slide 1
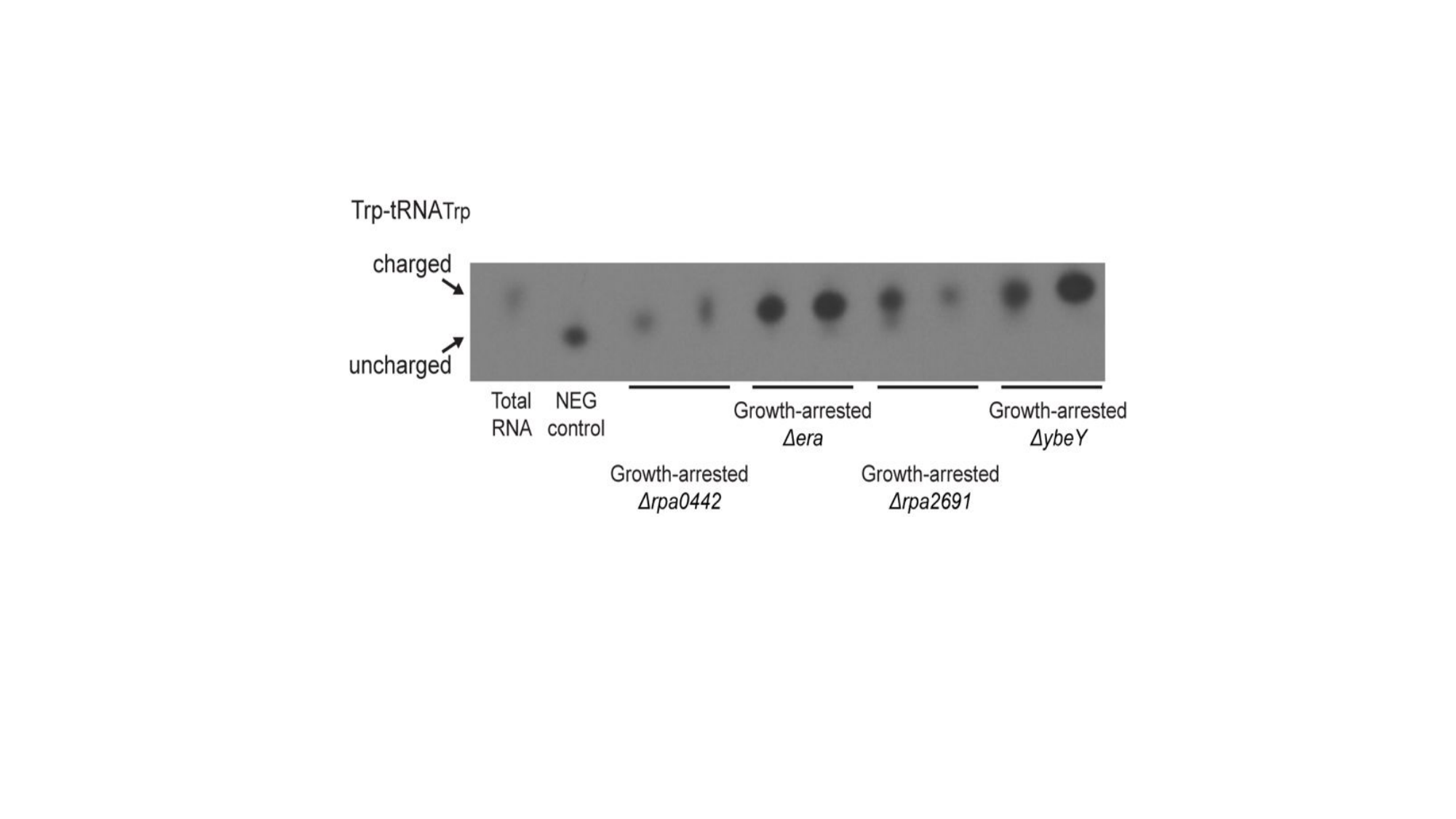

Supplement: FIG S4 [file mBio.02189-19-sf004.ppt]
